# Hypothermia with Extreme Bradycardia following Spinal Cord Infarction of Septic Origin

**DOI:** 10.1155/2017/1351549

**Published:** 2017-10-04

**Authors:** Philippe Hantson, Thierry Duprez

**Affiliations:** ^1^Department of Intensive Care, Cliniques Universitaires St-Luc, Brussels, Belgium; ^2^Department of Neuroradiology, Cliniques Universitaires St-Luc, Brussels, Belgium

## Abstract

Among other autonomic dysfunctions complicating acute spinal cord injury, deep hypothermia is rare but may induce serious cardiovascular complications. There are few pharmacological options to influence hypothermia. A 66-year-old woman was transferred to the intensive care unit (ICU) for serious cardiac arrhythmias (atrial fibrillation and asystole) in the context of a deep hypothermia (axillary temperature below 32°C). She had been admitted to the hospital two months before for an acute L4-L5 infectious spondylodiscitis without any initial neurological deficit. After surgery for epidural abscess drainage, she became paraplegic due to spinal cord infarction (from C7 to T6 levels) in the upper territory of the anterior spinal artery. In the ICU, the patient experienced several episodes of asystole and hypotension associated with a core body temperature below 35°C. Common causes of hypothermia (drugs, hypothyroidism, etc.) were excluded. A definitive pacemaker had to be inserted, but hypotension persisted. The prescription of oral progesterone (200 mg·d^−1^) helped to maintain a core temperature higher than 35°C, with a withdrawal of vasopressors. This case report illustrates that patients with incomplete spinal cord injury may present with delayed and deep hypothermia leading to serious cardiovascular complications. Progesterone could be able to influence positively central and peripheral thermal regulation.

## 1. Introduction

Autonomic dysfunction complicating spinal cord injury (SCI) may consist in cardiac arrhythmias, orthostatic hypotension, and thermoregulatory disturbances. Deep hypothermia around 32°C may increase the risk for severe cardiocirculatory complications [1, 2]. We describe a case of deep hypothermia complicating spinal cord infarction due to discal/intracanalar sepsis. Critically low core temperature was significantly improved by oral progesterone.

## 2. Case Presentation

**Figure 1:**
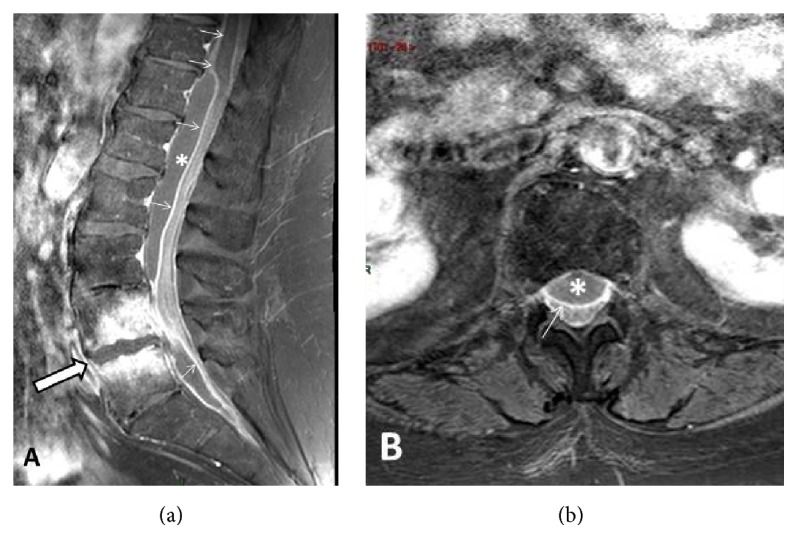
Lumbar spine MR work-up at admission. (a) Midsagittal contrast-enhanced (CE) T1-weighted view with Fat Suppression (FS) option. Spondylodiscitis at L4-L5 level (arrow) with necroticocystic intersomatic abscess and strong enhancement of the adjacent bone marrow. Epidural abscess extending from T11/T12 to S1/S2 was well seen (asterisk) together with intense enhancement of the dura (arrows). (b) Axial–transverse CE T1-weighted view through the L1 level. Anterior epidural abscess is well seen (asterisk) impinging on posteriorly displaced thecal sac. Note intense enhancement of the dura (arrow).

A 66-year-old woman with a previous medical history of hyperthyroidism and chronic myeloid leukaemia developed an acute L4-L5 infectious spondylodiscitis a few days after having received dental care. She complained from low back pain but had no neurological deficit. The initial magnetic resonance imaging (MRI) (Figure 1(a)) demonstrated a necroticocystic lumbar spondylodiscitis complicated by huge epidural abscess with diffuse inflammation of adjacent meninges.

**Figure 2:**
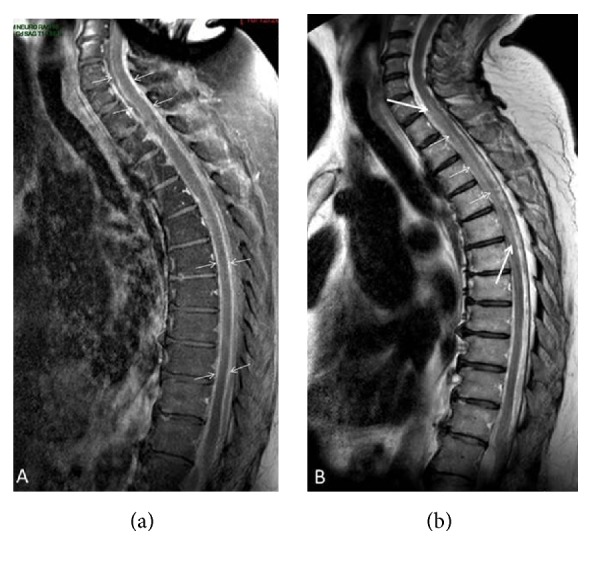
Follow-up MR examination of the cervicodorsal segments. (a) Midsagittal CE T1-weighted view showing strong enhancement of both dura and pia (between arrows) surrounding spinal cord. (b) Midsagittal T2-weighted view showing relative hypersignal intensity within upper anterior spinal artery territory between T1/T2 and T5/T6 disks (arrows) corresponding to spinal cord infarction.

**Figure 3:**
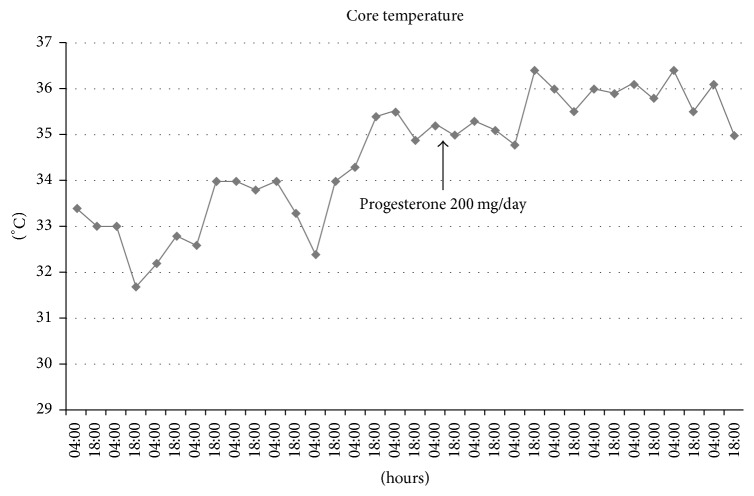
Core temperature recorded from a central venous catheter twice a day (4:00 and 18:00), before and after the introduction of oral progesterone.

The patient underwent surgical exploration and resection of the L4-L5 intervertebral disc. The cerebrospinal fluid (CSF) examination revealed 396 white blood cell count/*μ*l, glucose 31 mg·dl^−1^, proteins 366 mg·dl^−1^, and lactate 4.3 mmol·l^−1^. Intraoperative CSF culture returned positive for* Prevotella loescheii*. In the immediate follow-up of the surgical procedure, the patient was found paraplegic with also anaesthesia at a T6-T7 level. A follow-up spinal cord MRI (Figure 1(b)) revealed the onset of a spinal cord infarction from C7 to T6 in the upper territory of the anterior spinal artery featured by a sharply delineated hypersignal intensity within involved areas on T2-weighted images (Figure 2). While staying in the rehabilitation unit, the patient developed atrial fibrillation that was treated by sotalol. Profound hypothermia (32.2°C axillary) was recorded for the first time four days later, together with a low arterial blood pressure (58/39 mmHg) and a lowered consciousness. Echocardiography revealed a marked and diffuse depression of the left ventricular function. There was also a recent worsening of renal function with oliguria. The patient was transferred to the intensive care unit (ICU) for persisting hypotension, and marked hypothermia (32°C) was confirmed. The patient even presented a transient episode of asystole. With the suspicion of drug accumulation, sotalol therapy was stopped. Hemodynamic conditions gradually improved after dobutamine infusion and the patient returned to the rehabilitation unit. She was readmitted in the ICU one month later with asystole and was again successfully resuscitated. Hypothermia was confirmed by the recording of the core temperature by a central venous thermodilution catheter. Core temperature always remained below 35°C. The decrease in body temperature was never followed by shivering, but well by an alteration of consciousness. Several episodes of hypotension with extreme bradycardia (heart rate below 30 min^−1^) and even transient asystole were recorded when the temperature dropped to 32-33°C. Pharmacological causes of drug-induced hypothermia could be excluded. Noteworthily, a significant weight loss and skeletal muscle wasting were noted over the last weeks. Thyroid function tests were within the normal range. Brain MRI was unremarkable. External rewarming techniques (blankets, increase of room ambient temperature) remained unsuccessful. Due to the recurrence of extreme bradycardia with episodes of sinus block, a definitive pacemaker had to be inserted; after that a coronary angiography had failed to reveal coronary lesions. Sudden hypotensive episodes still occurred and norepinephrine infusion needed to be continuously adapted. Hypotension occurred frequently during the night shift, at a time that core temperature was lower. In an attempt to rise core temperature, oral progesterone 100 mg was prescribed twice per day (Figure 3). A significant difference (Student's *t*-test, *p* < .0001) was noted for the mean core temperature recorded during the eight days before and after progesterone therapy. Progesterone transient withdrawal for two days was followed by a new drop in core temperature. The maintenance of a core temperature above 35°C allowed stopping norepinephrine infusion. After the start of progesterone, there was no clear evidence for orthostatism when the patient was mobilized from her bed to a chair during the day shift. The neurological status of the patient also significantly improved and was ultimately discharged from the ICU. At follow-up, two months later, left ventricular function had also improved at echocardiography.

## 3. Discussion

Abnormal thermal regulation in SCI is mainly due to reduced sensory input to temperature regulation centres and to the loss of sympathetic control of temperature and sweat control below the level of injury [3–7]. Hypothermia produces effects on many organ systems, including hemodynamic instability with arrhythmias and depression of myocardial contractility [2]. While subnormal temperatures may be recorded in patients with spinal cord injury, critically low temperature in the hypothermic range (<35°C) was found in only 25/867 (3% of the patients) measurements obtained from 50 patients with chronic tetraplegia [8]. In a study enrolling patients with incomplete SCI, core and body temperatures (measured by digital infrared thermographic imaging) were analysed among three groups: SCI patients with neurological injury level at T6 or above, SCI patients at T7 or below, and control subjects [9]. While there was no statistically significant difference in the comparison of core temperatures among the three groups, patients with upper SCI had lower skin temperature than the patients with lower SCI and control group. This confirms the role of peripheral receptors in body temperature control in patients with upper SCI [5]. Body surface temperature is affected by diverse factors but is mainly controlled by the sympathetic nervous system. Cold exposure stimulates cold-sensitive receptors present in the skin, leading to prompt vasoconstriction that preserves heat dissipation and stimulates shivering reaction that increases basal metabolic rate and heat production. Investigations in paraplegic patients have shown that these patients will start to shiver to increase heat production at a lower central temperature than control subjects [5, 6]. Otherwise, a reduced peripheral vasoconstrictive response to adrenergic stimuli is likely to contribute to orthostatic tolerance and to an increase in heat dissipation. In healthy women, it has been shown that progesterone (200 mg·d^−1^) enhanced the cutaneous vasoconstrictor responsiveness in women with normal/high orthostatic tolerance, an effect that seems to be mediated by cyclooxygenase [10, 11]. Whether this concept could be applied to reduce orthostatism or heat dissipation from peripheral vasodilation was never investigated in patients with complete or incomplete SCI. Additionally, progesterone may also tend to shift central regulation of body temperature to higher temperature [12].

Finally, the influence of progesterone on thermal regulation was rarely investigated in men. The daily administration of 10 mg progesterone to six men resulted in a significant rise in temperature with a significant decrease in skin conductance [13].

## 4. Conclusion

Profound hypothermia with extremely serious cardiovascular manifestations may also be encountered in paraplegic patients. In the present observation, the extension of the ischemic medullar injury to a high-level probably explained that a large proportion of the skin was insentient, with an impaired response to changes in cold and hot. The apparent benefit of progesterone therapy could be ascribed to either a central (higher set point) or peripheral (increase of cutaneous vasoconstriction) mechanism, but this has to be confirmed by other observations as prolonged progesterone therapy may also induce side effects.
